# Effect of Load on Quartz Crystal Microbalance Sensor Response Addressed Using Fractional Order Calculus

**DOI:** 10.3390/s23156768

**Published:** 2023-07-28

**Authors:** Ioan Burda

**Affiliations:** Physics Department, Babes-Bolyai University, 400084 Cluj-Napoca, Romania; ioan.burda@gmail.com

**Keywords:** QCM sensor, fractional order BVD model, fractional order calculus, impedance analyzer

## Abstract

To accurately model the effect of the load caused by a liquid medium as a function of its viscosity, the fractional order Butterworth–Van Dyke (BVD) model of the QCM sensor is proposed in this study. A comprehensive understanding of the fractional order BVD model followed by a simulation of situations commonly encountered in experimental investigations underpins the new QCM sensor approach. The Levenberg–Marquardt (LM) algorithm is used in two fitting steps to extract all parameters of the fractional order BVD model. The integer-order electrical parameters were determined in the first step and the fractional order parameters were extracted in the second step. A parametric investigation was performed in air, water, and glycerol–water solutions in ten-percent steps for the fractional order BVD model. This indicated a change in the behavior of the QCM sensor when it swapped from air to water, modeled by the fractional order BVD model, followed by a specific dependence with increasing viscosity of the glycerol–water solution. The effect of the liquid medium on the reactive motional circuit elements of the BVD model in terms of fractional order calculus (FOC) was experimentally demonstrated. The experimental results demonstrated the value of the fractional order BVD model for a better understanding of the interactions occurring at the QCM sensor surface.

## 1. Introduction

Fractional order calculus (FOC) is a generalization of ordinary differentiation to the non-integral case and has become an extremely useful tool in applied mathematics and physics in recent decades [1,2]. Many physical phenomena exhibit intrinsic behavior that can only be explained using the FOC approach. In addition, in many applications, FOC describes the behavior of physical systems better and more accurately than traditional models [3,4,5,6].

Impedance spectroscopy is an efficient method for determining the electrical impedance of devices under test (DUT) or complex materials [7,8]. An electrical stimulus in the form of a current or voltage, such as a sinusoidal signal, chirp signal, or noise signal, is applied to a DUT to determine its electrical impedance by measuring the response to excitation. The impedance, Z(jω), is calculated using the ratio of voltage to current at the end of the measurement procedure [9,10].

The frequency-dependent losses in conventional circuit elements serve as the basis for the development of fractional order circuit element theory [11,12,13]. This theory was additionally expanded to include memristive components (memristor, memcapacitor, and meminductor) [14,15]. A resistance (R), a fractional order inductance ($L_{\alpha }$), and a fractional order capacitance ($C_{\beta }$) are used as fractional order circuit elements to introduce fractional order impedance for all possible topologies. As a result, a fractional order topology has three ordinary RLC variables and two additional fractional order variables, α and β. This fundamental generalization of impedance adds a new dimension to the modeling of electrical circuits [16,17].

However, many complex physical phenomena or nonlinear systems can be modeled with equivalent electrical circuit elements of fractional order $RL_{\alpha }C_{\beta }$. It should be noted that identical results cannot be produced using ideal RLC circuit elements. The performance obtained is always better than that of conventional integer calculus due to the additional degrees of freedom provided by the selection of fractional order circuit elements [7,18]. In addition, modeling using FOC provides more generalized and fundamental information extraction [19,20,21,22]. Additionally, by fitting additional parameters and applying an appropriate optimization approach, the output can be adjusted to be consistent with the experimental results.

An AT-cut quartz crystal is set up as a quartz crystal microbalance (QCM sensor) with electrodes on both sides. When an alternating electrical field is applied, the QCM sensor oscillates in the thickness shear direction [23,24]. Sauerbrey’s innovative research demonstrated that mass loading caused a change in the serial resonant frequency of the QCM sensor [25]. Surface mechanical impedance can be used to represent the mass load acting on the surface of the QCM sensor. For the equivalent electrical circuit, the mechanical impedance was changed to the electrical impedance. The static capacitance, $C_{p}$, is connected in parallel with the series motional branch that contains the circuit elements $R_{m}$, $L_{m}$, and $C_{m}$ to form the Butterworth–Van Dyke (BVD) model of the unloaded QCM sensor [26,27]. In the BVD model, $R_{m}$ represents energy dissipation (related with the damping factor), $L_{m}$ represents inertial mass, and $C_{m}$ represents mechanical elasticity. The ideal circuit elements $R_{m}L_{m}C_{m}$ of the series branch motional impedance in parallel with the static capacitance $C_{p}$ model the behavior of the QCM sensor only around resonant frequencies [28].

As long as measurements are performed in vacuum or air, the BVD electrical model of the QCM sensor assures a perfect impedance fit around resonant frequencies. This scenario, described by Sauerbrey’s equation [25,26], is not affected by coating one or both electrodes of the QCM sensor with a thin sensing layer if it is firmly bound, and its mass does not change the linear dependence of the serial resonant frequency with the deposited mass. In this situation, an experimental setup that consists of an oscillator with a QCM sensor in the loop [29] and a frequency meter is commonly used. This experimental configuration might be more efficient if the parallel capacitance $C_{p}$ was compensated to eliminate the discrepancy between the serial resonant frequency of the QCM sensor and the frequency of the oscillator output. Unfortunately, this difference also depends on the damping induced by the working medium and details of the electronic oscillator [30].

The modeling of interactions occurring at the QCM sensor surface as well as experimental methods [31,32,33] have undergone significant changes owing to the expansion of applications in liquid media [34,35,36]. The viscosity of the liquid also causes a change in the serial resonance frequency and damping factor; therefore, at least these parameters of the QCM sensor must be measured simultaneously. Finally, the data obtained refer to the mass of the material deposited on a single electrode of the QCM sensor as well as its viscoelastic properties.

The determination of all electrical parameters of the BVD model is a recent trend due to the technological advances in impedance analyzers [9,37]. The impedance analyzer uses a passive approach; therefore, under ideal conditions, the electrical parameters of the BVD model or the derived physical parameters of the QCM sensor are unaffected [35]. In reality, the experimental setup and topology for adapting the QCM sensor to a general-purpose impedance analyzer are sources of measurement uncertainty [38].

In this study, the behavior of the reactive motional circuit elements of the BVD model was examined from the viewpoint of FOC in relation to the viscosity of the liquid medium covering one of the QCM sensor electrodes. The main contributions of this study are (i) a theoretical study of the BVD model with fractional order reactive motional circuit elements, (ii) a simulation of commonly encountered cases in experimental impedance spectroscopy of QCM sensors, (iii) the development of an advanced method for processing raw experimental data based on two-step successive fitting to determine the electrical parameters of the fractional order BVD model, and (iv) an experimental evaluation of the fractional order effects on the reactive motional circuit elements of the BVD model in liquid media with different viscosities.

The remainder of this paper is organized as follows. After a brief introduction, the fractional order BVD model is detailed in Section 2. This section concludes with a simulation of the frequency response of a QCM sensor using a fractional order BVD model and an experimental setup involving a virtual impedance analyzer (VIA). In Section 3, the benefits of the fractional order BVD model and the experimental outcomes of impedance spectroscopy augmented by VIA compensation using a FOC-based approach in air [38] are presented. Section 4 and Section 5 present the final discussion and the conclusions, respectively.

## 2. Materials and Methods

### 2.1. Fractional Order Reactive Circuit Elements

Modern manufacturing techniques for capacitors and inductors largely ensure integer-order behavior, thus excluding any fractional order effects in BVD electronic circuit design. Supercapacitors and lossy coils are examples of capacitors and inductors exhibiting fractional order properties [11,39]. Rigorous analysis of real circuit elements often does not confirm integer-order behavior. In general, FOC provides a better description of the behavior of real capacitors and inductors.

In the continuous time domain, the fractional order derivative operator is defined by the Riemann–Liouville or Caputo definitions; in the discrete time domain, the Grunwald–Letnikov definition is used [40,41]. In terms of fractional order, the Caputo [42] derivative operator for *α* > 0 is defined as:

(1) Dtα0Cft=1Γn−α∫0t(t−u)n−α−1ddunfudu, n−1<α<n∈R+

where $\Gamma \left(\cdot \right)$ denotes Euler’s gamma function. The fractional derivative in the sense of Caputo is represented by the Laplace transform as follows:

(2) L[Dtα0Cf(t)]=sαF(s)−∑k=0n−1sα−k−1f(k)(0)

Considering, in Equation (2), the particular situation 0<α<2, it follows:

(3) LDtα0Cft=sαFs−sα−1f0, 0<α<1

(4) LDtα0Cft=sαFs−sα−1f0−sα−2f′0, 1<α<2

The relationship between voltage, $v_{L}(t)$, and current, $i_{L}(t)$, for fractional order inductance, is expressed as follows:

(5) $$v_{L}\left(t\right)=L_{\alpha }\frac{d^{\alpha }}{dt^{\alpha }}i_{L}(t)$$

where α (0<α<2) is the fractional order of the inductance denoted $L_{\alpha }$ and the fractional order derivative operator is represented by Dtα0C=dα/dtα [16]. Similarly, the relationship between the current $i_{C}(t)$ and voltage $v_{C}(t)$ in the case of the fractional order capacitance is as follows:

(6) $$i_{C}\left(t\right)=C_{\beta }\frac{d^{\beta }}{dt^{\beta }}v_{C}(t)$$

where $C_{\beta }$ is the fractional order capacitance of the order β (0<β<2). The phase difference is (π/2)α for fractional order inductance and (π/2)β for fractional order capacitance. For each reactive circuit element, the fractional order cannot be greater than two without undergoing a transformation into the conjugate complex circuit element.

If the current through the inductance at the initial time $i_{L}(0)=0$ and the DC voltage across the capacitance $v_{C}(0)=0$, using the Laplace transformation on both sides of Equations (5) and (6) the following is obtained:

(7) $$V_{L}\left(s\right)=L_{\alpha }s^{\alpha }I_{L}\left(s\right)$$

(8) $$I_{c}\left(s\right)=C_{\beta }s^{\beta }V_{C}(s)$$

The following formula can be used to determine the fractional order impedance of the inductance and capacitance in the s-domain:

(9) $$Z_{L_{\alpha }}\left(s^{\alpha }\right)=\frac{V_{L}\left(s\right)}{I_{L}(s)}=s^{\alpha }L_{\alpha }$$

(10) $$Z_{C_{\beta }}\left(s^{\beta }\right)=\frac{V_{C}\left(s\right)}{I_{C}(s)}=\frac{1}{s^{\beta }C_{\beta }}$$

where, considering s=jω, it is easy to calculate the fractional order impedance of the reactive circuit elements.

### 2.2. Fractional Order BVD Model

**Figure 1:**
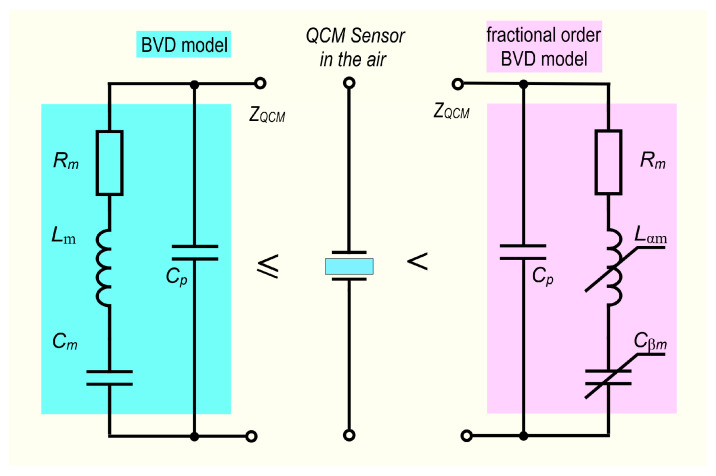
The BVD and the fractional order BVD model.

The unavoidable existence of parallel capacitance, $C_{p}$, in the BVD model is correctly criticized from a theoretical and experimental standpoint [24,28]. In the case of a real capacitor, fractional order behavior is typically determined by electrode roughness, surface disorder, slowdown of adsorption or diffusion reactions, electrode shape, and electrode porosity. Because parallel capacitance is easily compensated for in an impedance analyzer, this situation is not the subject of this study. In this investigation, only the reactive motional circuit elements were regarded as having a fractional order. Figure 1 illustrates both BVD model versions examined in this study using theoretical analysis, simulations, and experiments.

The vital role of the reactive motional circuit elements in simulating the behavior of the QCM sensor supports this choice. From the perspective of the FOC, the motional resistance, which is of integer-order, explains the dissipative processes of the QCM sensor. The s-domain impedance of the fractional order BVD model is described by the following equation:

(11) $$Z(s^{\alpha ,\;\beta })=\frac{Z_{s}{\left(s^{\alpha ,\;\beta }\right)Z}_{p}(s)}{Z_{s}(s^{\alpha ,\;\beta })+Z_{p}(s)}$$

The impedance of the motional branch is determined by the following relation when utilizing the fractional order BVD model of the QCM sensor and considering s=jω:

(12) $$Z_{s}(j\omega^{\alpha ,\beta })=R_{m}+(j{\omega )}^{\alpha }L_{\alpha m}+\frac{1}{{\left(j\omega \right)}^{\beta }C_{\beta m}}$$

The generalization of the BVD model to the fractional order BVD model yields a new relation for the impedance of the motional branch expressed in Equation (12). Two new parameters, *α* and *β*, were added to this model to provide additional versatility when modeling the behavior of the QCM sensor.

### 2.3. Fractional Order BVD Model Simulation

The behavior of the fractional order BVD model can be clearly observed by evaluating Equation (11) in the resonant frequency range. If parallel resonance is present, it is necessary to consider the parallel capacitance, $C_{p}>0$ to ensure consistency with the experimental results obtained using an impedance analyzer. The impedance of the QCM sensors at the serial resonant frequency and parallel resonant frequency are of crucial importance for determining the electrical parameters of the BVD model.

**Figure 2:**
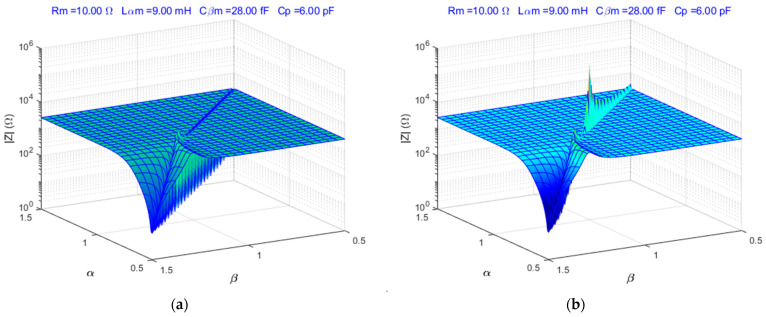
Fractional order BVD model: (**a**) serial resonances, (**b**) parallel resonances.

The simulation scripts of the fractional order BVD model were developed in MATLAB^®^. The fractional order BVD model was simulated for 0.5<α<1.5, 1.5>β>0.5, and typical QCM sensor parameters. Figure 2 shows the simulation results for the fractional order BVD model in the resonant frequencies range.

The response of the fractional order BVD model changes significantly at parallel resonance when the parallel capacitance $C_{p}=6\;pF$ is considered, as illustrated in Figure 2b. The FOC is unique when compared to integer-order calculus, where the aforementioned surfaces are compressed to a single point (α=β=1).

**Figure 3:**
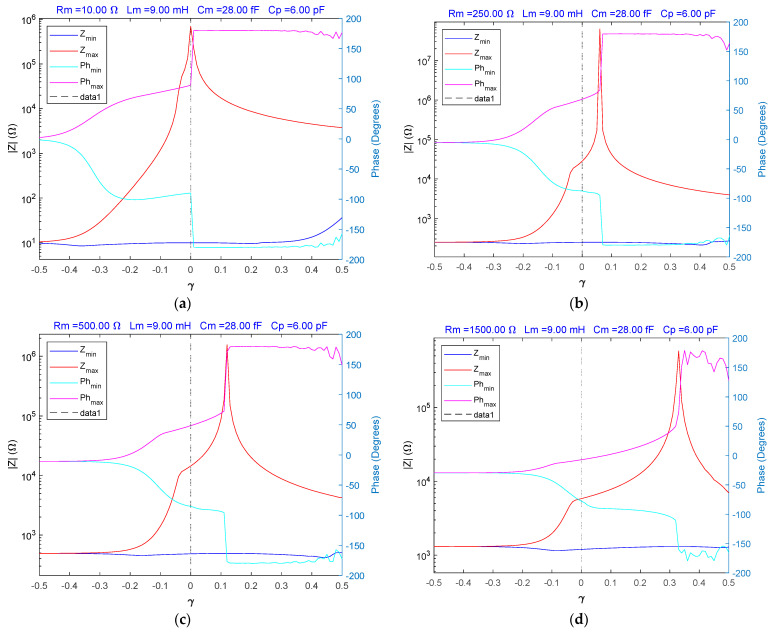
The fractional order BVD model for γ in the range −0.5 to 0.5S: (**a**) $Z_{min}$, $Z_{max}$ impedance and phase for $R_{m}=10\;\Omega$, (**b**) $Z_{min}$, $Z_{max}$ impedance and phase for $R_{m}=250\;\Omega$, (**c**) $Z_{min}$, $Z_{max}$ impedance and phase for $R_{m}=500\;\Omega$, (**d**) $Z_{min}$, $Z_{max}$ impedance and phase for $R_{m}=1500\;\Omega$.

Equation (12) can be simplified to the following form from the simulation presented above to demonstrate the helpfulness of the fractional order BVD model:

(13) $$Z_{s}\left(j\omega^{\left(1\pm \gamma \right)}\right)=R_{m}+(j{\omega )}^{\left(1+\gamma \right)}L_{\alpha m}+\frac{1}{{\left(j\omega \right)}^{\left(1-\gamma \right)}C_{\beta m}}\;$$

where α=1+γ and β=1−γ considering the interval −0.5<γ<0.5. The results of the simulation of the fractional order BVD model are shown in Figure 3.

Depending on the parameters γ and $R_{m}=\{10,\;250,\;500,\;1500\}$ in Figure 3, the minima and maxima of the impedance and phase of the fractional order BVD model of the QCM sensor are considered.

### 2.4. Bode and Nyquist Plot of the Fractional Order BVD Model

**Figure 4:**
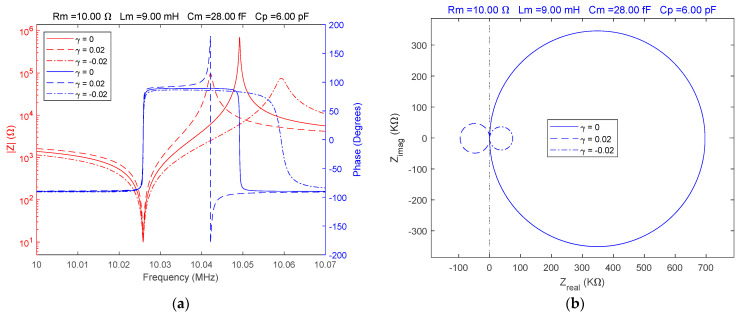
Fractional order BVD model for $R_{m}=10\Omega$ and γ=[0, 0.02,−0.02]: (**a**) Bode plot, (**b**) Nyquist plot.

In experimental studies using impedance spectroscopy, it is necessary to investigate the response of the QCM sensor around the resonant frequencies using Bode and Nyquist plots. First, considering a typical situation for a QCM sensor located in air, a reference for impedance spectroscopy was established. The Bode and Nyquist plots illustrate the response of the QCM sensor in this case. If α=β=1 or equivalently, γ=0, the fractional order BVD model is equivalent to the standard BVD model. The values of the electrical parameters of the fractional order BVD model were chosen to be equivalent to the experimental parameters of a QCM sensor with a serial resonance frequency of about 10 MHz. The behavior of the QCM sensor was simulated for values of γ=[0, 0.02,−0.02] and the results are shown in Figure 4.

Without changing the electrical parameters of the fractional order BVD model, the QCM sensor response is significantly modified by apparently insignificant fractional order values, as shown in Figure 4a,b.

**Figure 5:**
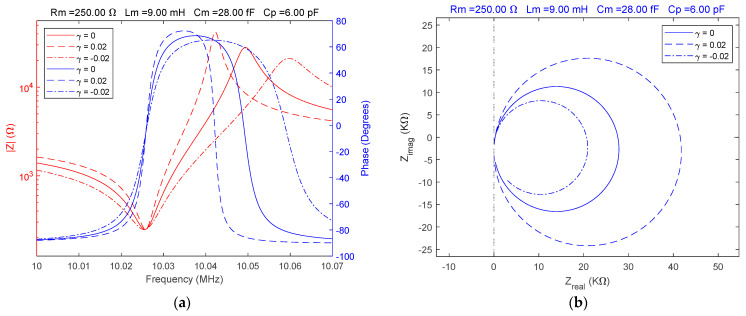
Fractional order BVD model for $R_{m}=250\;\Omega$ and γ=[0, 0.02,−0.02]: (**a**) Bode plot, (**b**) Nyquist plot.

A substantial change in the behavior of the QCM sensor is caused by the transition from air to a liquid medium as illustrated in Figure 5.

In this case, a water-specific damping was modeled using the value of the motional resistance ($R_{m}=250\;Ω$) without taking into account the change in other electrical parameters that would only cause a shift in resonant frequencies.

Considering the damping specific to a liquid medium the effects of fractional order values different from the ideal case are illustrated in Figure 5 and allow a comparative evaluation of the QCM sensor response. A significant difference in the spectral positions of the resonance frequencies between the two fractional order cases relative to the ideal case (γ=0) was observed. This difference is extremely useful for a more accurate modeling of the QCM sensor response in liquid media.

**Figure 6:**
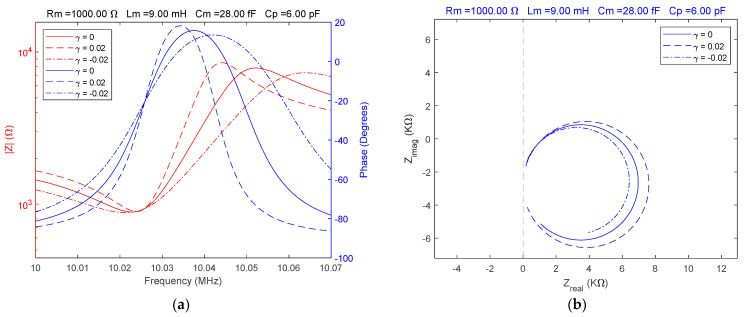
Fractional order BVD model for $R_{m}=1000\;\Omega$ and γ=[0, 0.02,−0.02]: (**a**) Bode plot, (**b**) Nyquist plot.

The complex behavior of QCM sensors in various high-viscosity liquid media has been extensively studied [26,27,35]. Such a situation is unsatisfactorily modeled by the BVD model in many experimental situations, especially when the electrical properties of the liquid medium make it difficult to interpret the experimental data from the perspective of an acoustic or mechanoelectric model. Based on this viewpoint, the adaptability of the fractional order BVD model adds a new dimension to the assessment of experimental data. A high value of the motional resistance $R_{m}=1000\;Ω$ was considered for the simulation. In order to make a comparative evaluation, three situations identical to the ones presented above were considered. The simulation results of the fractional order BVD model in the case γ=[0, 0.02,−0.02] are illustrated in Figure 6.

The spectral position of the parallel resonance frequency is generally accepted to be a function of the parallel capacitance, $C_{p}$, which is also responsible for its existence. In experimental practice, compensation for the parallel capacitance of the QCM sensor is theoretical; in reality, any residual value is a substantial source of error in liquid media with high viscosity. This is true even with the aid of an impedance analyzer [28]. Because the fractional order value also models the location of the parallel resonant frequency in the Bode plot, the fractional order BVD model offers a new perspective.

The versatility and effects generated by the fractional order BVD model of the QCM sensor have been demonstrated by simulating situations frequently encountered in experimental practice. The advantages of modeling the QCM sensor response using the fractional order BVD model were also investigated experimentally using VIA.

### 2.5. Virtual Impedance Analyzer and Experimental Setup

Generalized impedance analysis is now used in many sensor applications owing to advancements in electronic technology [23,43]. The impedance analysis approach for the QCM sensor was based on passive interrogation to evaluate its response in the frequency domain. The impedance analysis method, from the viewpoint of the BVD model of the QCM sensor, ensures the exploration of its behavior at a level that can only be accomplished using this method [28,37].

The compensation techniques used by the impedance analyzer have a significant impact on the performance of impedance spectroscopy. The typical compensation process [9,28] starts by compensating for the open-circuit parasitic impedance, $Z_{oc}$, and then moves on to compensate for the short-circuit parasitic impedance, $Z_{sc}$. The following equation is used to determine the corrected value of the impedance, $Z_{Q}$, of the QCM sensor:

(14) $$Z_{Q}=\frac{Z_{rm}-Z_{sc}}{1-(Z_{rm}-Z_{sc})/Z_{oc}}$$

where $Z_{rm}$ is the raw measured impedance of the QCM sensor.

**Figure 7:**
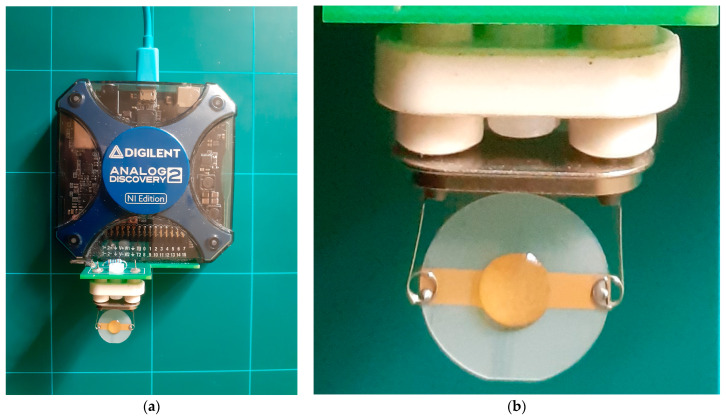
Experimental setup: (**a**) virtual impedance analyzer (VIA), (**b**) QCM sensor with a drop of glycerin–water solution on top of it.

The core advantages of digital technology include the ability to develop high-precision equipment with automatic data processing capabilities, provide automatic calibration, and enhance data transfer capabilities through high-speed interfaces. During the data acquisition stage, we used an impedance analyzer to measure the electrical impedance of the QCM sensor around the resonance frequencies. The QCM sensor response was entirely defined by computing the electrical parameters of the BVD model based on raw experimental data [9,28]. The literature [9,28] contains detailed discussions of the electrical properties and functionality of the virtual impedance analyzer (VIA) employed in the experimental research reported here. Figure 7a shows the use of an Analog Discovery 2 (AD2) virtual instrument from Digilent Inc. (Pullman, WA, USA) [44] and an external shield with minimal complexity.

The hardware resources required to build an impedance analyzer with a decent performance were provided by the AD2 virtual instrument. Two analog input channels and two analog output channels were present. When analyzing the technical capabilities of the AD2, it is important to consider the electrical performance of the ADC and DAC converters with a resolution of 14 bits at 100 MSPS. They were digitally synchronized to measure the phase difference. To produce the virtual instrument, additional hardware resources were incorporated into a Xilinx Spartan 6 FPGA (XC6SLX16-1L) [44]. As shown in Figure 7b, a measuring cell was not used to reduce the impact of mechanical stress on the QCM sensor.

## 3. Results

This section presents extensive experimental studies that support the fractional order BVD model of a QCM sensor in liquid media. Figure 7 shows the experimental setup of a QCM sensor with a fundamental resonance frequency of approximately 10 MHz (151225-10, International Crystal Manufacturing Co., Inc., Oklahoma City, OK, USA). The following VIA configuration was used to acquire the raw experimental data: (i) passive excitation in the resonant frequency range with a sine wave of 1 V amplitude and (ii) measurement of the QCM sensor’s impedance and phase at 50,001 points. A thermally isolated container was used to evaluate the QCM sensor response, reducing external interference with the experimental setup. The experiment was fully automated using a Python application, and the experimental data were processed in real time, creating a robust measurement setup for validating the fractional order BVD model.

### 3.1. VIA Compensation Using the Fractional Order BVD Model

**Figure 8:**
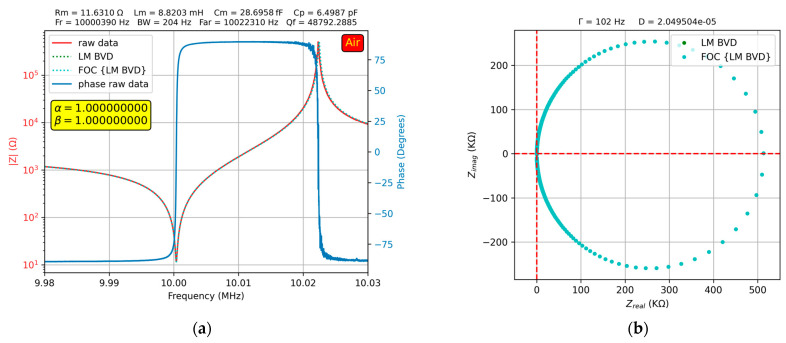
Fractional order BVD model for the QCM sensor in air: (**a**) Bode plot, (**b**) Nyquist plot.

The experimental study in air involved the following steps: (i) BVD compensation of the VIA [28] in air followed by verification of the compensation using the fractional order BVD model [38]; (ii) determination of the BVD parameters in air based on the experimental resonance frequencies [28]; and (iii) using the Levenberg–Marquardt (LM) algorithm to fit the BVD model and fine-tune the raw electrical parameters. To evaluate the fractional order effect, the value of the electrical parameters of the previously determined BVD model was considered, and a rerun with the fractional order BVD model was performed. Again, the LM algorithm was used, but only fractional order parameters were used for the fit. The results of the procedures described above in the referenced case when the QCM sensor was in air are shown in Figure 8.

As shown in Figure 8a, the use of the fractional order BVD model was not justified because α=β=1 in this case. Moreover, this has been demonstrated in [38], in which the evaluation of VIA compensation using the FOC was investigated. To achieve a high accuracy in the optimal VIA compensation procedure, it is recommended to use the raw electrical parameters determined based on the experimental resonance frequencies without refining them using the LM algorithm. In this situation, the obtained values are very close to the integer-order value, but are slightly affected by experimental uncertainty. The high-accuracy experimental results allow us to conclude that the QCM sensor does not exhibit fractional order behavior in air as long as the VIA is optimally calibrated because the Nyquist plot in Figure 8b shows a perfect overlap between the two BVD models.

### 3.2. Fractional Order Effect Induced by Load on QCM Sensor Response

In accordance with the BVD procedure, in which it is assumed that the value of the motional capacitance, $C_{m}$, does not change in the liquid medium and that the frequency shift is due to the change in the motional inductance, $L_{m}$, the fractional order BVD model is investigated experimentally. It should be noted that in a liquid medium, the value of the motional resistance $R_{m}$ also changes as a function of viscosity. For a systematic evaluation of the QCM sensor from the perspective of the fractional order BVD model, glycerol–water solutions were prepared in 10% increments, and boundary situations with only water or glycerol were evaluated.

**Figure 9:**
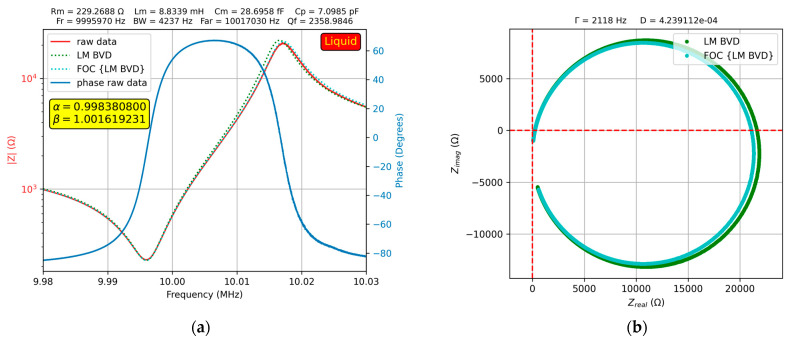
Fractional order BVD model for the QCM sensor in water: (**a**) Bode plot, (**b**) Nyquist plot.

The results of the experimental investigations in water are shown in Figure 9a, where a fractional order effect of the QCM sensor can be observed, providing a better fit to the experimental data.

In addition, in Figure 9b, the Nyquist plot shows the difference in fit between the BVD model and fractional order BVD model. The measurement procedure was the same as that in air, and the value of the motional capacitance, $C_{m}=28.6958\;fF$, was kept constant in liquid media according to standard BVD methodology.

The resonant frequency shift of the resonant frequency in the liquid medium is modeled using the motional inductance ($L_{m}$). In liquid medium the motional resistance ($R_{m}$) changes due to its viscosity as well as the motional inductance ($L_{m}$) due to the attached mass and associated properties induced by its stiffness. A change in parallel capacitance ($C_{p}$) was observed due to the electrical properties of the liquid medium. To better fit the experimental data, we considered in a second step that $L_{m}$ and $C_{m}$ could be of fractional order and performed the calculations again, taking into account the values previously determined by fitting them with the fractional order BVD model. The values of $L_{m}$, $R_{m}$, and $C_{p}$ were determined with the standard BVD model and then fractional order parameters (α and β) were calculated for a better fit. It was observed that the presence of the liquid medium changes (not much) the ideal character of the motional inductance and capacitance.

**Figure 10:**
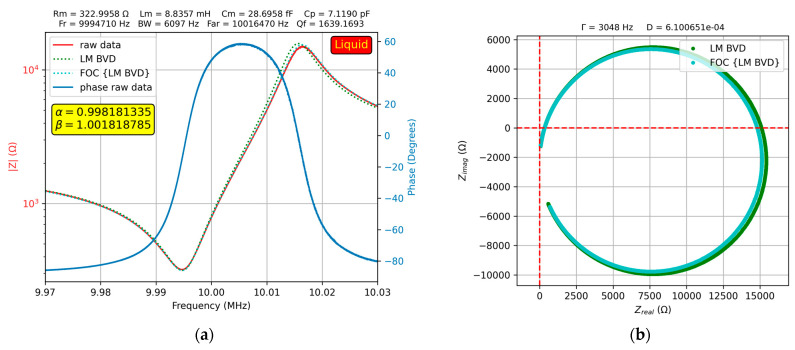
Fractional order BVD model for the QCM sensor in 20% glycerin–water solution: (**a**) Bode plot, (**b**) Nyquist plot.

Figure 10a shows the results of the experimental investigations on the QCM sensor, where a 20% glycerin–water solution was deposited on one of the electrodes.

As expected, in this case, the fit of the fractional order BVD model was better than that of the BVD model. This difference is highlighted in the Nyquist plot in Figure 10b. For some glycerol–water solutions, the results of the experimental investigations are presented using four samples to provide insight into the advantages offered by the fractional order BVD model from an experimental perspective.

**Figure 11:**
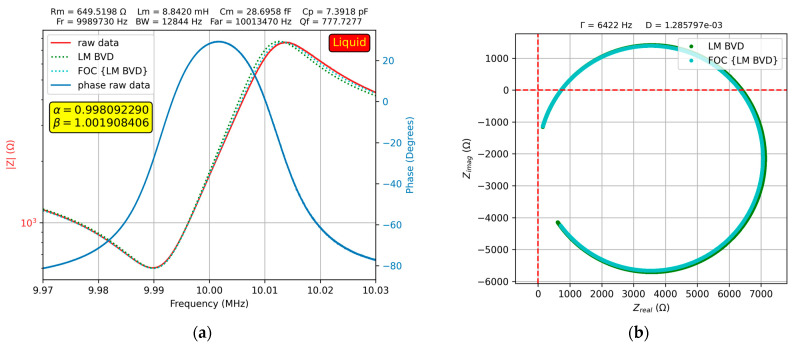
Fractional order BVD model for the QCM sensor in 50% glycerin–water solution: (**a**) Bode plot, (**b**) Nyquist plot.

The results of the experimental investigation of a 50% glycerol–water solution is shown in Figure 11a.

**Figure 12:**
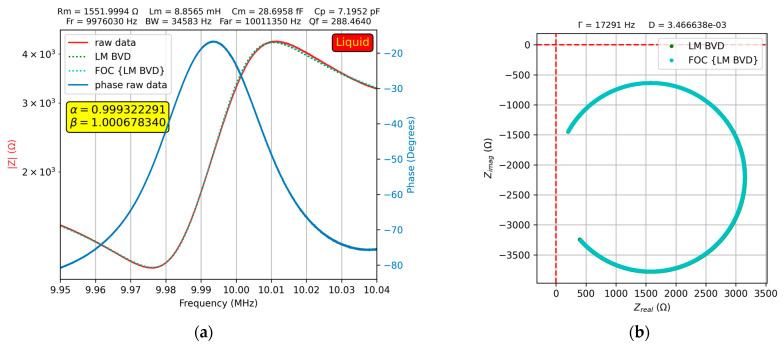
Fractional order BVD model for the QCM sensor in 90% glycerin–water solution: (**a**) Bode plot, (**b**) Nyquist plot.

At high glycerol–water solution concentrations, both the fractional order BVD model and BVD model fit the experimental data well. Figure 12 shows this result for a 90% glycerol–water solution.

**Figure 13:**
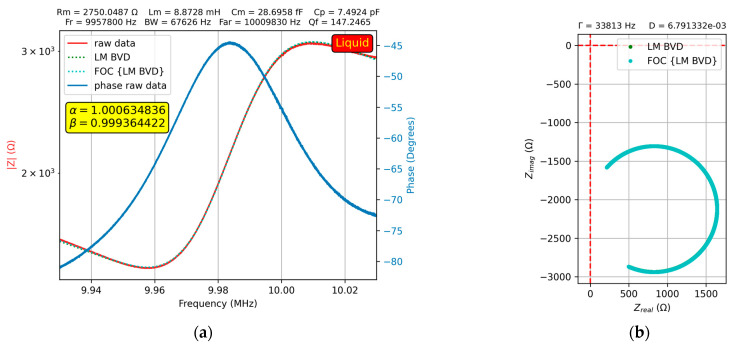
Fractional order BVD model for the QCM sensor in glycerin: (**a**) Bode plot, (**b**) Nyquist plot.

This may be due to the increased dissipation process reflected by the high value of motional resistance. This is also reflected in Figure 12b, where the real axis no longer intersects. Another explanation is the high stiffness of the glycerol–water solution at high concentrations, which leads to a different behavior of the QCM sensor. This claim was confirmed by the experimental investigation of the fractional order BVD model, where the droplet deposited on one of the QCM sensor electrodes was pure glycerol. The results of the experimental investigation are shown in Figure 13.

**Figure 14:**
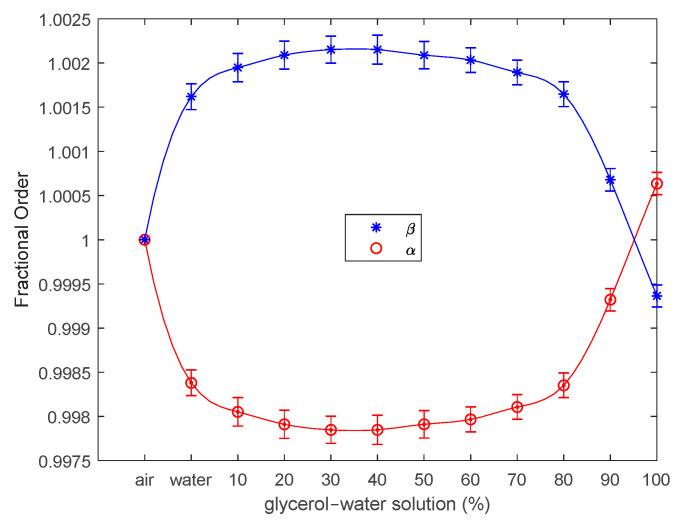
Fractional order parameters of the QCM sensor in air, water, and glycerin–water solutions.

In this case, both BVD models fit the experimental data very well, with a particularity regarding the value of the fractional order parameters, where α>β in the case of pure glycine. An overview of the evolution of the fractional order parameters with respect to the working medium is shown in Figure 14.

As shown in Figure 14, the fractional order effect of the liquid working medium on the QCM sensor was experimentally demonstrated. For each experimental situation investigated, five measurements were performed, the experimental errors were negligible (error bars), and there was no effect induced by experimental uncertainty or thermal drift. In each case investigated experimentally, the fit of the experimental data to the fractional order BVD model was better than that to the BVD model. Apparently, the fit is acceptable for both models at glycerol–water solution concentrations greater than 80%. This is owing to the high value of the motional resistance, $R_{m}$, as well as a decrease in the fractional order parameter values, as illustrated in Figure 14.

## 4. Discussion

Fractional order circuit elements allow for more precise modeling of the nonlinear behavior of the QCM sensor. The reinterpretation of the BVD model with fractional order circuit elements, $L_{m}$ (fractional order inductance) and $C_{m}$ (fractional order capacitance), can provide some advantages and address certain aspects of QCM sensor behavior in QCM technology. Fractional order elements can be used to model memory effects on the behavior of the QCM sensor. This can contribute to a more comprehensive description of changes in the frequency response of QCM sensors [24,26,35]. In addition, reinterpreting the BVD model with fractional order circuit elements can lead to improved precision and accuracy in QCM measurements, as it allows for a more detailed description of the actual behavior of the QCM sensor.

Fractional order parameters highlight that the effect of the liquid medium on the QCM sensor is unexpected, but has been experimentally demonstrated. Even though the deviation from integer-order for reactive motional circuit elements is not major (Figure 14), the contact between one of the QCM sensor electrodes and liquid media of different viscosities induces a response specific to fractional order reactive circuit elements. It is easy to intuit the diversity of particular situations induced by various liquid media, especially in the field of biosensors based on QCM sensors.

Two distinct applications of FOC in QCM technology have been highlighted. The first application presented in detail in the literature [38] is essentially a highly sensitive method for verifying the optimal compensation of an impedance analyzer. By compensating the VIA using the FOC method any fractional order behavior for the reactive motional circuit elements of the BVD model is eliminated. A fractional order behavior of the reactive motional circuit elements was induced by an incorrect VIA compensation. As long as the QCM sensor is in the air, a behavior consistent with the integer-order BVD model is unanimously accepted [33]. An important aspect to investigate experimentally is presented in Section 3.2 and illustrates the behavior of the QCM sensor, in liquid media of different viscosities, from the FOC perspective. Otherwise, a second application is presented in this study and consists in experimentally investigating the behavior of the QCM sensor as a function of load using the fractional order BVD model. By using fractional order circuit elements, a better fit of the experimental data and the evolution of their associated fractional order parameters as a function of the interactions occurring at the QCM sensor surface was confirmed, providing additional information.

## 5. Conclusions

A large spectrum of QCM sensor behavior can be covered by the FOC according to extensive simulations of the fractional order BVD model. An advantage in experimental practice is the ability to evaluate the α and β parameters of the fractional order BVD model and determine whether the VIA compensation is accurate [38]. A comprehensive understanding of the fractional order BVD model, followed by simulation of commonly encountered situations in experimental practice, provides the necessary support.

Experimental evidence of the impact of fractional order on the reactive motional circuit elements of the BVD model is presented. For the fractional order BVD model, a parametric analysis was performed in air, water, and glycerol–water solutions in ten-percent steps. This study showed that when the QCM sensor was switched from air to water, its behavior changed significantly, and that this change followed a specific dependence on the viscosity of the glycerol–water solution.

The experimental findings demonstrate the capacity of the fractional order BVD model to improve the comprehension of the interactions occurring on the QCM sensor’s surface. Impedance spectroscopy evaluation of the fractional order BVD model highlights the benefits of FOC for simulating complicated phenomena that are frequently encountered in experimental investigations.

## Data Availability

No Data for this article.
